# Human adenovirus associated with severe cold agglutinin syndrome: a rare complication in Pediatrics

**DOI:** 10.1590/1984-0462/2024/42/2022174

**Published:** 2023-07-10

**Authors:** Julia Loureiro Sion, Angelica Lucía Hidalgo Flores, Regina Aparecida Cardoso, Marlene Pereira Garanito

**Affiliations:** a Universidade de São Paulo, São Paulo, Brazil.

**Keywords:** Autoimmune hemolytic anemia, Adenovirus, Pediatrics, Anemia hemolítica autoimune, Adenovírus, Pediatria

## Abstract

**Objective:**

The objectives of this study were to describe the first pediatric case of cold agglutinin syndrome (CAS) triggered by human adenovirus and review the literature.

**Case description:**

This case report involves a previously healthy, 2½-year-old female child with human adenovirus isolated in a nasal swab. At 72 h after admission, the patient progressed to a severe episode of anemia (hemoglobin level: 2.6 g/dL). The laboratory findings were consistent with CAS. The patient received blood transfusion, vitamin supplementation, adequate hydration, and thermal protection. At her last follow-up, 1 year after her initial presentation, she remains clinically well without signs of hemolysis.

**Comments:**

While severe CAS is extremely uncommon in the pediatric emergency department, human adenovirus infection is a common illness in pediatrics. Recently, the adenovirus has been associated with new complications (acute hepatitis and fulminant liver failure). Pediatric physicians and hematologists should be aware of unusual evolution, signs, and symptoms of this infection that warrant more urgent medical attention. In this case, the hematologic complication suspicion was the key to early diagnosis and adequate management.

## RESUMO

**Objetivo:**

Descrever o primeiro caso pediátrico de síndrome da crioaglutinina desencadeado por adenovírus humano e revisar a literatura.

**Descrição do caso:**

Paciente do sexo feminino, dois anos e seis meses, previamente hígida e diagnosticada com adenovírus humano isolado em *swab* nasal. Após 72 horas da admissão, a paciente evoluiu com quadro de anemia grave (hemoglobina de 2,6 g/dL). Os achados laboratoriais foram compatíveis com síndrome da crioaglutinina. A paciente recebeu transfusão de concentrado de hemácias, suplementação vitamínica, hidratação adequada e proteção térmica. Em seu último retorno ambulatorial, um ano após a apresentação inicial, permanecia clinicamente bem, sem sinais de hemólise.

**Comentários:**

Enquanto a síndrome da crioaglutinina grave é extremamente incomum na emergência pediátrica, a infecção por adenovírus humano é um quadro comum na infância. Recentemente, o adenovírus tem sido associado a novas complicações, e pediatras e hematologistas devem ficar atentos à possibilidade de uma evolução incomum dessa infecção e dos sinais e sintomas que possam necessitar de atenção urgente. No caso apresentado, a suspeita da complicação hematológica foi a chave para o diagnóstico precoce e seu manejo adequado.

## INTRODUCTION

Cold agglutinins were first described by Landsteiner in 1903 and may be seen with the cold agglutinin disease, paroxysmal nocturnal hemoglobinuria, and cold agglutinin syndrome (CAS).^1,2^

The term CAS is used for secondary cold-agglutinin-mediated autoimmune hemolytic anemia (AIHA) occasionally complicating specific infections or malignancies. The classic CAS with infectious etiology typically involves adolescents with *Mycoplasma pneumoniae* or Epstein-Barr virus, but it has also been described with other agents such as cytomegalovirus (CMV), influenza, varicella, hepatitis C, rubella, mumps, and measles virus.^3,4^

The overall annual incidence of AIHA is reported to be approximately 0.2 per million individuals under 20 years, and CAS has been reported to account for about 20–25% of AIHA.^3^

To the best of our knowledge, we report the first pediatric case of a CAS triggered by human adenovirus (HAdV) and review the literature.

## CASE REPORT

A previously healthy, 2-½ year-old female child was admitted to the emergency department with fever (40°C), rhinorrhea, decreased appetite, poor fluid intake, and somnolence for 4 days. At the physical examination, she had signs of dehydration, blood pressure 109x71 mmHg, heart rate 119 beats/min, respiratory rate 34/min, oxygen saturation 97%, and axillary temperature 36.5°C. The laboratory examination showed hemoglobin (Hb) 11.6 g/dL, white blood cell count 13,270/mm^3^, platelets count 175,000/mm^3^, and normal chest X-ray. HAdV was isolated in a nasal swab using real-time polymerase chain reaction (RT-PCR). She received intravenous fluid to correct the dehydration and stayed in the hospital for observation and continuous monitoring.

At 72 h after admission, the patient developed vomiting, diarrhea, and dark urine, and the blood tests showed a rapidly progressing anemia (Hb level decreased from 11.6 to 2.6 g/dL). The serum level of lactate dehydrogenase (LDH) was 2,582 U/L (normal range: 120–203 U/L), indirect bilirubin level 2.08 mg/dL (normal range <0.6 mg/dL), haptoglobin <10 mg/dL (normal range: 30–200 md/dL), and 0.15% (7,400/mm^3^) reticulocyte count. Her direct antiglobulin test (DAT) was positive, attributable to complement (C3d); the indirect antiglobulin test (4°C) detected cold agglutinin in the serum with red cell agglutination 4+/4. A review of the peripheral smear revealed red blood cell (RBC) auto-agglutination and Rouleaux formation. The presence of hemoglobin in the urine was positive. Serological tests for Epstein-Barr virus (EBV), CMV, hepatitis B, C, HIV, measles, and herpes simplex 1 and 2 were negative. Nasal swabs using RT-PCR for coronaviruses (i.e., 2019-nCoV/SARS-CoV-2, NL63, 229E, OC43, and HKU-1); influenza viruses A [FluA], A[H1N1], and B; human bocavirus; parainfluenza viruses 1, 2, 3, and 4; human metapneumoviruses A and B; rhinovirus; respiratory syncytial viruses A and B; human parechovirus (HPeV); and *M. pneumoniae* were negative. Fecal rotavirus and adenovirus were excluded. Cerebrospinal fluid samples were collected and tested for HAdV, HPeV, herpes viruses (1, 2, 6, and 7), EBV, CMV, varicella zoster virus, parvovirus B19 (B19V), and enterovirus using multiplex PCR assay, and the results were negative. The sample was tested for the B19V DNA using RT-PCR, and B19V was not detected. Bacterial and fungal infections were not identified.

Autoimmune diseases, primary immune deficiency syndromes, hematologic disorders, lymphoproliferative diseases, and solid malignancy were excluded.

The patient received an RBC transfusion (15 mL/kg/day for 2 days), folic acid, oral vitamin B12, adequate hydration, and thermal protection. Following these interventions, the hemoglobin began to stabilize (10 g/dL), the patient began to clinically improve, and she was discharged on day 14 of hospitalization. As a preventive measure, the patient was recommended to avoid cold exposure.

**Figure 1.**
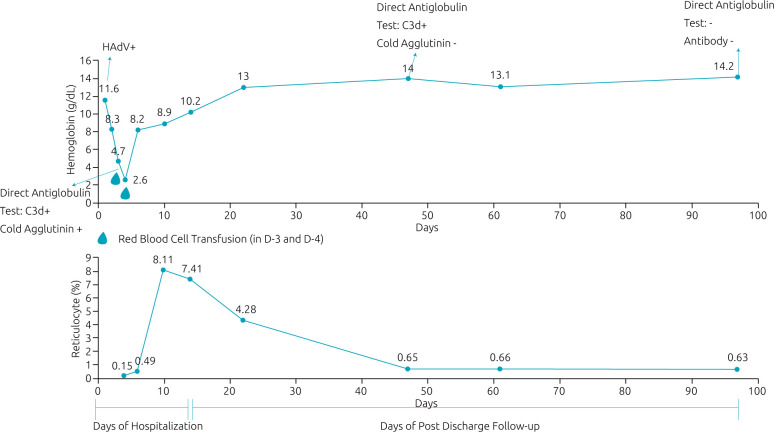
Hemoglobin level, reticulocyte, direct antiglobulin test, and cold agglutinin trend throughout hospitalization and post-discharge follow-up.

Approximately 1½ months after admission, the patient’s Hb, LDH, and reticulocyte count were normal. DAT and the serum antibody screen became negative 3 months after infection. Figure 1 illustrates her hemoglobin and reticulocyte trend from her admission until normalization.

At her last follow-up, 1 year after her initial presentation, she remains clinically well without signs of hemolysis, despite other acute respiratory infections caused by HAdV.

## DISCUSSION

A case of severe CAS is extremely uncommon in the pediatric emergency department. To the best of our knowledge, this case is the first description of a child that had no known underlying malignancy or infectious disease typically associated with CAS.

Pediatric CAS is usually abrupt, short in duration, and self-limited. The onset of anemia occurs during the second or third week after the infection has started, and even though the hemolytic anemia can be severe, the prognosis is generally good. The hemolysis decreases as the infection clears up and completely resolves within 4–10 weeks.^2,4^

In CAS, the cold agglutinins (IgM autoantibodies) may lead to intravascular and extravascular hemolysis. The autoantibodies (IgM) bind RBC membrane antigens, typically of the erythrocyte I/i antigen system, in the cold (4°C), although they may maintain reactivity up to ≥30°C, defined as a wide thermal amplitude. IgM binds C1q on the red cell membrane and fixes complement components, and complement cascade activation occurs. This process leads to the extravascular removal of C3b-coated red cells by the reticuloendothelial system; however, exacerbations can activate C5, leading to intravascular hemolysis. The cleaved product C3d is detected by DAT, which is characteristically positive for only C3d, although a weak positive IgG is sometimes also detected. The pentavalent IgM structure is able to bind simultaneously multiple RBCs resulting in agglutination and the appearance of RBC Rouleaux on the blood smear.^2,5,6^ Spherocytes are absent because of predominantly intravascular hemolysis.^7^

The clinical presentation involves non-specific signs and symptoms of jaundice, dark urine, fatigue, splenomegaly, and possibly hepatomegaly. Additionally, acrocyanosis may occur with exposure to cold. The primary laboratory evaluation is similar for all forms of pediatric AIHA. The diagnosis is defined as the presence of hemolytic anemia, a positive polyspecific DAT result, a monospecific DAT result positive for C3d only, and a cold agglutinin titer ≥64 at 4°C. Cold agglutinins are present in a small percentage of the general population, and not all patients who develop cold agglutinins following infection will develop clinically significant hemolysis. Titers <64 are clinically benign and not leading to hemolysis.^2,4,8,9^

Our patient presented with reticulocytopenia at the time of diagnosis. In AIHA, reticulocytopenia is a rare presentation (20%). It might reflect an immune-mediated destruction or autoantibody-induced apoptosis of RBC precursors within the bone marrow, a temporary suppression of bone marrow activity secondary to infection, and/or reflect a lag in marrow responsiveness to the hemolytic event.^3,6^ The increase in reticulocytes occurred after transfusion.

Until now, no evidence-based therapy exists for the CAS secondary to infection. Prospective trials or well-designed retrospective series have not been published, and all recommendations are based on case reports and clinical experience.^4^ A primary goal is to treat the underlying infection, if possible. For example, if *M. pneumoniae* is detected as the etiology of the infection, specific treatment should be instituted with antibiotics. Useful measures include non-pharmacological management: keeping the patient warm/normothermic (in particular, the head, face, and extremities); infusion of cold liquids should be avoided; if transfused, the packed RBCs should be warmed; the extremity chosen for infusion should be kept warm, and the use of an in-line blood warmer is recommended; maintaining adequate hydration; and monitoring urine output in the presence of significant intravascular hemolysis.^3,4,10^ Transfusion, when indicated, can be considered safe, and in most cases, compatibility problems are generally not encountered in CAS. It is usually easy to find compatible donor erythrocytes, and screening tests for irregular blood group antibodies are most often negative.^4,5^

In critical situations where it is not feasible to wait for the resolution of infection, plasmapheresis may be helpful as a temporizing measure in selected cases, in the acute phase. The theoretical rationale for this procedure is strong because both the sensitized RBCs and the circulating autoantibodies can be removed simultaneously, and quickly, but no prospective study has been published and some conflicting data do exist.^7^ The recommendations have been to exchange 1–1.5 times the plasma volume with albumin, not plasma, daily or every other day. Once complement proteins can exacerbate hemolysis, transfusion of blood products with a high plasma content should probably be avoided.^4,5^ Corticosteroid therapy or other immunosuppressive medications are not indicated, and splenectomy would not be beneficial as hemolysis occurs mostly intravascularly.^2,7^

In this report, we described a rare hematologic complication of a common illness in pediatrics. To the best of our knowledge, there is no evidence in the literature of healthy pediatric patients presenting CAS secondary to HAdV. Once HAdV infection is currently associated with new complications, especially acute hepatitis and progression to fulminant liver failure, pediatric physicians and hematologists should be aware of unusual evolution, signs, and symptoms of this infection that warrant more urgent medical attention.^11,12^ The high degree of hematologic complication suspicion is the key to early diagnosis and adequate management.

## Data Availability

The database that originated the article is available with the corresponding author.

## References

[1] Chou ST, Schreiber AD, Orkin SH, Nathan DG, Ginsburg D, Look AT, Fisher DE, Lux SE (2015). org. Nathan and Oski’s Hematology of infancy and childhood.

[2] Voulgaridou A, Kalfa TA (2021). Autoimmune hemolytic anemia in the pediatric setting. J Clin Med..

[3] Ladogana S, Maruzzi M, Samperi P, Perrotta S, Del Vecchio GC, Notarangelo LD (2017). Diagnosis and management of newly diagnosed childhood autoimmune haemolytic anaemia. Recommendations from the Red Cell Study Group of the Paediatric Haemato-Oncology Italian Association. Blood Transfus..

[4] Berentsen S, Tjønnfjord GE (2012). Diagnosis and treatment of cold agglutinin mediated autoimmune hemolytic anemia. Blood Rev..

[5] Berentsen S (2020). New insights in the pathogenesis and therapy of cold agglutinin-mediated autoimmune hemolytic anemia. Front Immunol..

[6] Hill A, Hill QA (2018). Autoimmune hemolytic anemia. Hematology Am Soc Hematol Educ Program..

[7] Kim TO, Despotovic JM (2019). Primary and secondary immune cytopenias: evaluation and treatment approach in children. Hematol Oncol Clin North Am..

[8] Gabbard AP, Booth GS (2020). Cold agglutinin disease. Clin Hematol Int..

[9] Berentsen S, Randen U, Tjønnfjord GE (2015). Cold agglutinin-mediated autoimmune hemolytic anemia. Hematol Oncol Clin North Am..

[10] Teachey DT, Lambert MP (2013). Diagnosis and management of autoimmune cytopenias in childhood. Pediatr Clin North Am..

[11] Rabaan AA, Bakhrebah MA, Nassar MS, Natto ZS, Al Mutair A, Alhumaid S (2022). Suspected adenovirus causing an emerging hepatitis among children below 10 years: a review. Pathogens..

[12] Kelgeri C, Couper M, Gupte GL, Brant A, Patel M, Johansen L (2022). Clinical spectrum of children with acute hepatitis of unknown cause. N Engl J Med..

